# Supplementary figures and images for: WormPose: Image synthesis and convolutional networks for pose estimation in C. elegans
Source: PLoS Comput Biol. 2021 Apr 27;17(4):e1008914. doi: 10.1371/journal.pcbi.1008914 (PMC8078761; doi:10.1371/journal.pcbi.1008914)

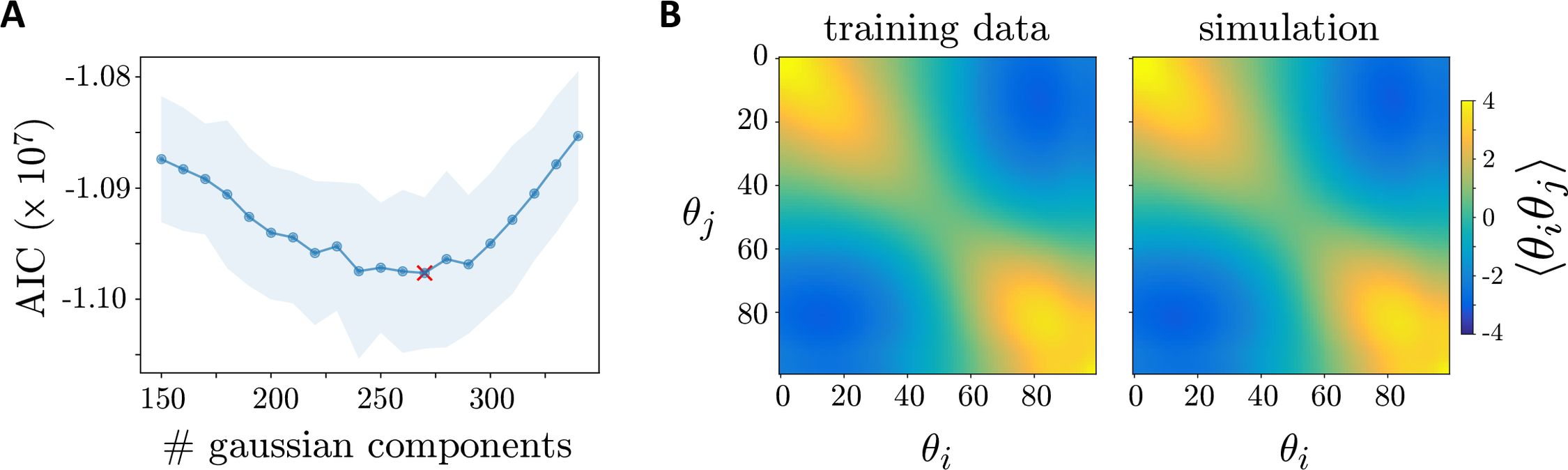

Supplement: S1 Fig — (A) Akaike Information Criterion for GMMs with different numbers of gaussian components. The minimum is attained with N = 270 gaussian components. Error bars represent 95% confidence intervals over 100 different training sets of ∼ 15000 worm shapes sampled uniformly according to the body curvature as measured by the third eigenworm coefficient, a3. (B) Covariance matrix of the space of mean subtracted tangent angles θ→ for the data used in training (left) and an equal number of simulated angles (right). (TIF) [file pcbi.1008914.s001.tif]

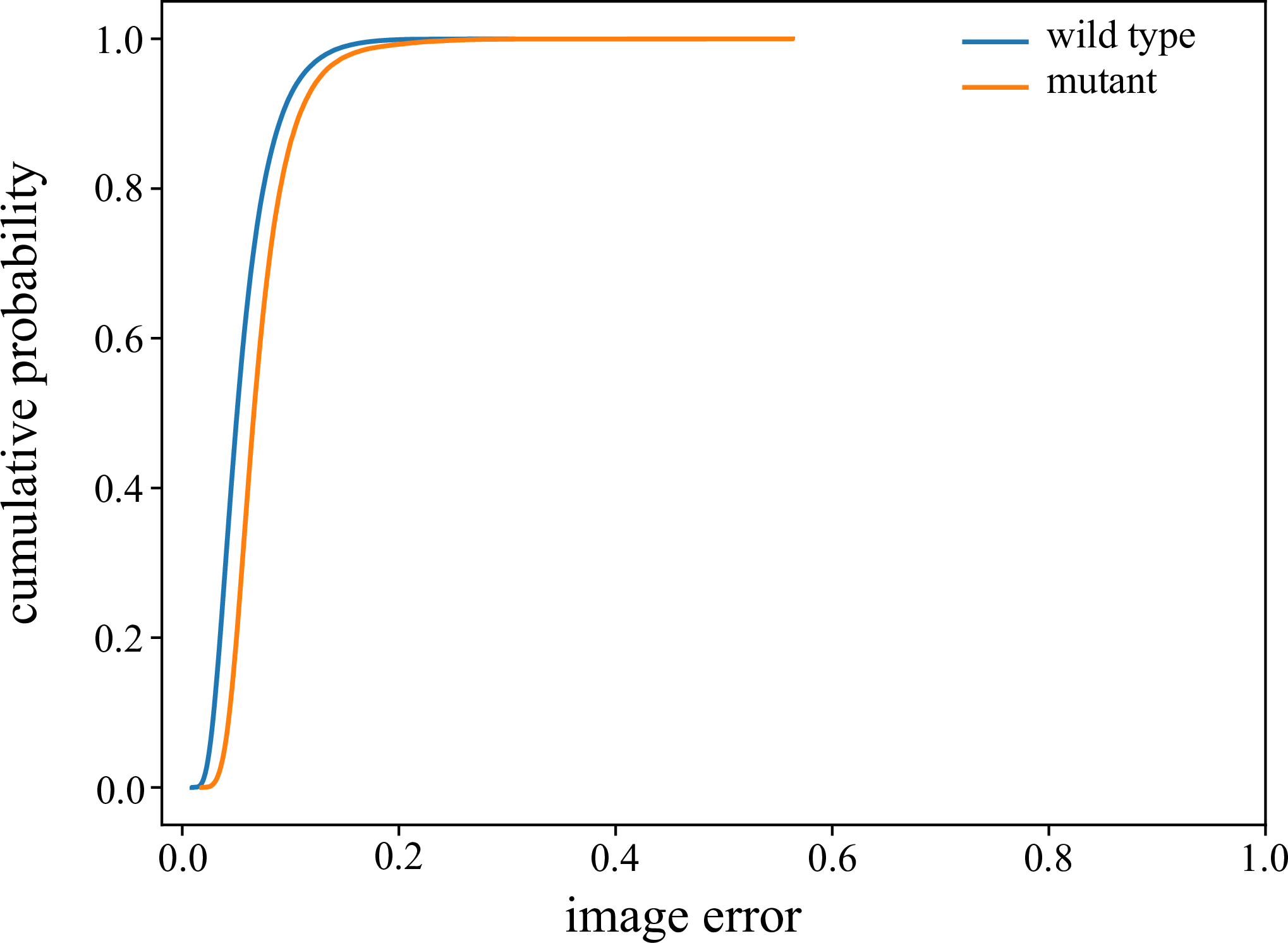

Supplement: S2 Fig — (TIF) [file pcbi.1008914.s002.tif]

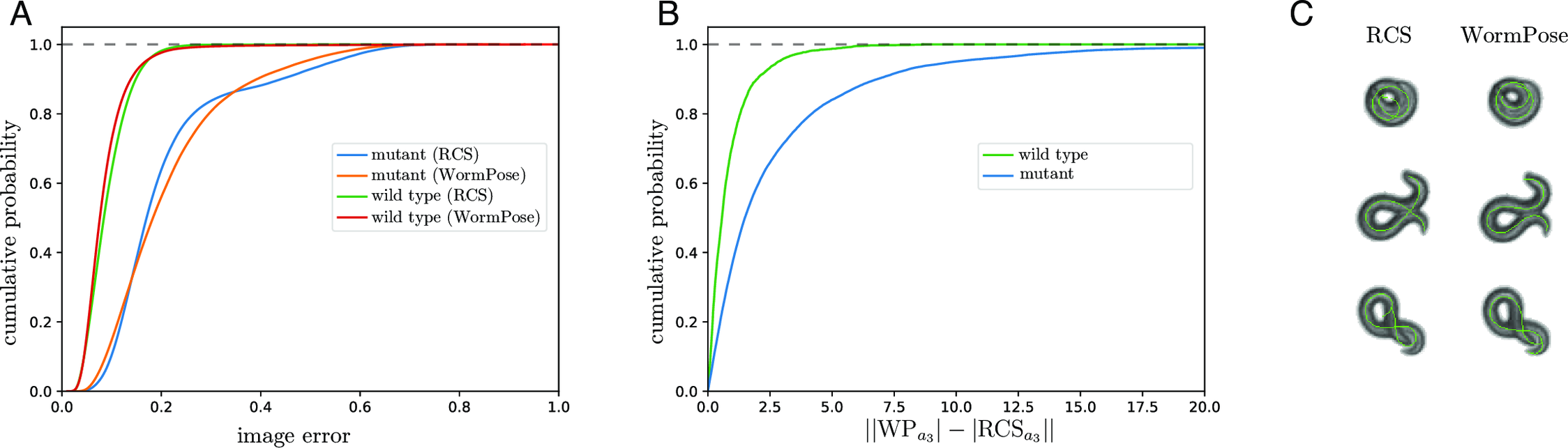

Supplement: S3 Fig — (A) We show the cumulative image error of predicted images, similarly to Fig 5A. While the image error is similar, WormPose is faster and does not make use of temporal information (a possible route for future improvement). (B) Cumulative distributions of the difference in a3 mode values δ=‖WPa3|-|RCSa3‖, restricted to coiled shapes (|a3| > 15) and image error ≤0.3 as determined from the output of WormPose. We plot separate distributions for the wild-type and mutant strains. Large deviations between the methods occur primarily in the coiled mutants and we manually examine a subset of 100 images with δ > 10 (a difference chosen to facilitate comparisons by eye) where we find 72% correctly tracked by WormPose, 6% correctly tracked by RCS, and 22% in which the better tracked centerline was unclear. A video of this inspection process is available with the data. (C) Qualitative results for a selection of frames where the image error doesn’t fully describe the discrepancies between the two methods. Very tight loops (top) are challenging for both methods and RCS typically misidentifies crossings where greyscale information would help (middle and bottom). (TIF) [file pcbi.1008914.s003.tif]
