# Supplementary material for: WormPose: Image synthesis and convolutional networks for pose estimation in C. elegans
Source: PLoS Comput Biol. 2021 Apr 27;17(4):e1008914. doi: 10.1371/journal.pcbi.1008914 (PMC8078761; doi:10.1371/journal.pcbi.1008914)
Supplement: Attachment — Submitted filename: WormPose_rebuttal.pdf [file pcbi.1008914.s004.pdf]

Dear Editor,

Thank you for your correspondence and we are grateful for the efforts of the editorial staff and the reviewers in these challenging times across the world. We appreciate the encouraging response from editor and the referees. We have revised our manuscript to address all of the main referee points, noting the changes in red. We provide a point-by-point rebuttal response below.

Reply to Reviewer #1

We thank the reviewer for their positive comments, close reading, and for especially acknowledging the yet unrealized importance of coiled worm postures as well as our efforts at quantifying the tracking results.

- *When discussing previous methods of solving the coiling problem Huang et al. (2006) J. Neurosci. Meth. should be cited as well.*

  Thank you, we have added the reference.

- *"CNNs are the foundation for recent, remark- able progress in markerless body point tracking [15–17], including worm posture [18, 19]. However, intensive la- beling requirements by human annotators, even if as- sisted by technology [20], as well as the ambiguity of which or exactly how many points to label, offer a bar- rier to the usefulness of CNNs in posture tracking and beyond." If I've understood correctly, the method in reference 18 also works without manually annotated images so the discussion should be modified to reflect that.*

  We have removed (Li et al) from the introduction (where it was misplaced) so that the reference occurs only in the last paragraph of the discussion in the context of the use of synthetic images.

- *In the network architecture and training section more detail on the CNN should be provided (type of CNN (ResNet), how many layers, neurons per layer, etc.)*

  We have added a new paragraph describing our network architecture and size as well as additional references.

- *Typo: "we set set the weights"*

  Thank you for your detailed reading, we have corrected the typo.

- *C. elegans not italicised in second paragraph of discussion.*

  We have fixed the italics.

Reply to Reviewer #2

We thank the reviewer for their detailed reading and constructive comments, especially on our computational approach and the relevant quantification of error.

Various error quantifications

- *We would like to see a quantification of errors but for ground truth centerlines in real data.*

  We appreciate and understand the perspective of the reviewer and have thought carefully about the relevant quantification of error. It is important to emphasize that the most accurate ground truth centerlines for crossed shapes come from the generative process used to create our synthetic data. This is a unique benefit of the shape image generator and we do not believe that having humans draw centerlines on images would confer any additional benefit. Human labelling is necessarily limited and subject to uncontrolled bias: detriments that result from the individuality of the labeler, the ambiguity of drawing the centerline itself, and from non-representative image selection. We believe that these negatives outweigh the positive aspects of labels on experimentally generated images. We have added analysis and error quantification as detailed below which further supports the accuracy of WormPose.

- *The use of pixel error is less interpretable and does not address the difficult problems this algorithm aims to solve. This is particularly important for cases like that shown in Fig. 1C, where the pixel error may be very small, but the posture error may be very large. Indeed, how often does this algorithm correctly distinguish between the two possibilities in Fig. 1C? We wonder whether there could be a direct and intuitive metric used for evaluation, for example the fraction of correctly annotated centerline crossings.*

  We agree that a centerline or mode error provides a direct performance measure, which is why we included such errors in Fig. 5 (now revised as detailed in the next rebuttal point into separate shape categories). To address the question of small pixel yet large centerline error, we use our synthetic dataset to show the cumulative errors in a_3, selected for coiled shapes $|a_3| > 15$ and images that pass our similarity score, Rebuttal Fig. 1. Only $\sim 5\%$ of these shapes have mode error $\delta a_3 > 2$, indicative of $\lesssim 10\%$ error in the centerline.

- *Related, the underlying data are effectively split into easy postures (straight motion) and very difficult postures (coiled and self-occluding). Thus, it seems important to the claims of the paper to characterize the algorithm error separately for these qualitatively different clusters of postures, as in the similar panel of Fig. 2F in related previous work (Broekmans et al).*

  In a revised Fig. 5 we have included separate mode error distributions for uncrossed and crossed shapes, where the later are identified by $|a_3| > 15$. The median mode errors are small in all cases.

- *Similarly, the comparison to previous work is incomplete. Figure S4 compares the pixel-wise errors between this work and a previous algorithm, but given that the scientific output of this algorithm is centerlines, a direct comparison of centerline error seems necessary. In the second results section the authors state "Unfortunately, a lack of ground truth posture sequences means that we cannot directly compare the posture estimates of RCS and WormPose." However, a smaller set of manual annotations can be generated to produce this comparison.*

  In a revised figure (now Fig. S3) we have included cumulative distributions of the difference in a_3 mode values $\delta = ||a_3|_{\mathrm{WP}} - |a_3|_{\mathrm{RCS}}|$, restricted to coiled shapes ($|a_3| > 15$) and image error ≤ 0.3 as determined from the output of WormPose. We plot separate distributions for the wild-type and mutant strains. Large deviations between the methods occur primarily in the coiled mutants and we manually examine a subset of 100 images with $\delta > 10$ (a difference chosen to facilitate comparisons by eye) where we find 72% correctly tracked by WormPose, 6% correctly tracked by RCS, and 22% in which the better tracked centerline was unclear. A video illustrating this manual inspection process is available with the data.

- *A different step of the algorithm also appears to require an additional error reporting: the head-tail discrimination module. While the methodology is well explained, the overall fraction of correctly annotated heads/tails is not reported.*

  Head-tail assignment is not the focus of WormPose and misassignments are uncommon. From all N2 worms, we drew frames with $|a_3| \geq 15$ and image error ≤ 0.3, such that no two drawn frames are closer than 20 frames apart so as not to over count any potential errors from the same series of coiled shapes. Upon manual inspection we found NO head-tail errors. Nevertheless, in future work we plan to develop a separate head-tail detection network so as to robustly handle lower-quality and smaller images.

Minor data notes

- *The authors state "we also expect that the labeled frames provide the head-tail position at regular intervals throughout the video". Is there a GUI or expected format for this input? Although this requirement leaves the following statement in the abstract technically correct "thus avoiding the need for human-labeled training", it should be noted more prominently that analyzing a new dataset is not completely automated.*

  For generality, we have chosen to state the necessary input of the head-tail positions but left the exact format up to the user. In the "Data Requirements" section we do mention Tierpsy tracker as reasonable source for such input and we have added a sentence clarifying this point.

- *Related, the authors assume "that the input data consists of videos of a single moving worm and that most of the non-coiled frames are analyzed beforehand", but Section Results/"Comparison with previous approaches" states that WormPose can be performed on a laptop. A sentence about whether the entire pipeline (i.e. including the required pre-analysis) can also be done on a laptop should be included*

  We could not locate any text in the manuscript which mentions a laptop. Nevertheless, the reviewer raises an interesting question. So,we tested WormPose on a high-performance laptop with a Intel i7-10875H CPU and a NVIDIA GeForce RTX 2070 SUPER GPU, on the same N2 dataset and parameters of the paper. The dataset generation and training the network took 6.5 hours, and predicting the full dataset of 600k+ frames took less than 2 hours. We also see no barrier to performing pre-analysis, such as with Tierpsy, on a laptop, thus making the full pipeline accessible. We have added comments at the end of the Implementation section reflecting these results.

Minor algorithm notes

- *The authors use a GMM to build their generative model. It is unclear why this choice was made. We assume this method was chosen because it is simple, fast, common, and generative. However, this should be explained.*

  We thank the reviewer for the opportunity to clarify this point. The assumption is indeed correct. The main goal is to obtain a model of the worm shape probability distribution, such that we can generate realistic worm shapes in a flexible manner with the obtained GMM. In that sense, we want the GMM to capture the underlying distribution sufficiently well, while allowing for a certain degree of variability: this can be tuned by changing the number of Gaussian components in the mixture model. We have clarified our description in the section "Generating Worm Shapes".

- *In Fig. S2A, the authors use AIC to determine the number of components of their GMM. However, two things are unclear: Why was AIC chosen and not cross-validation? What are the error bars on the plot? Although it does not seem to affect the results at all, a sentence or two of explanation would be appreciated.*

  The goal of the GMM is simply to obtain a generative model of worm shapes that is flexible enough to allow for variability, beneficial for network training. We did not perform cross-validation because we are not interested in a classification task, for which generalization would be important. In addition, because this is not a supervised learning task (e.g. regression or classification), there is no notion of ground truth to compare the obtained labels against. While we could devise a cross-validation procedure in which a given metric could be used to score the resulting in the test set, the obtained optimal number of clusters would depend on the chosen metric. Thus, we decided to use AIC as a simple probe for the trade-off between the model accuracy and the number of parameters, allowing the user to tune the number of Gaussian mixtures according to the desired variability. We have added a sentence explaining the error bars in Fig. S1: they result from sampling a training dataset of ~ 15000 worm shapes uniformly in curvature, and computing the AIC curves from 100 different training sets.

Minor other notes and typos

- *In the second paragraph of section Methods/"Processing Natural Images" the quotes are wrong in the phrase: The morphological operation "close"*

  Thank you for your detailed reading, we have fixed the quotes.

  We have corrected the grammar.

Other computational notes

- *We commend the authors for a well presented open-source package. In particular, it is excellent that the package is installable via pip and the requirements are included, with versions, in the repository.*

  Thank you!

- *We believe that the computational speed of this package is a very strong asset, and could be emphasized more as an important scientific contribution.*

  Thank you. We now emphasize the increased computational speed of WormPose in the last paragraph of the introduction

Reply to Reviewer #3

We thank the reviewer for their comments and for the opportunity to expand upon the generality of our approach.

- *Overall, I think the method is sound and useful, however I wonder about its domain of applicability. Since it's based on eigenmode projections, its not clear to me that it will work efficiently in cases where worms are no longer well captured by the eigenworms derived from observations of agar crawling. This might include worms in more complex environments. If they could show that, for instance, thrashing worms could still be reasonably well resolved, or mutants that display radically different mechanical properties, rolling mutants etc. could be well captured with this eigenworm driven training set that*

would be valuable. I'm not sure if that would just reflect that robustness of the CNN or if that would suggest that the eigenworms from agar are generic enough to be pushed into new territory. In terms of domain of application, what about other problems where linear shapes are bent into self-occluding shapes (e.g. snakes or plant roots)?

We thank the reviewer for raising this point. We note that even the low-dimensional "eigenworm" space originally derived from the behavior of wild-type worms on food-free agar plates is robust enough to describe the on-food behavior of a wide (307 strains!) range of mutants with similar accuracy (Brown et al, *Proc Nat Acad Sci (USA)*, **110** 791-796 (2013)). Even this relatively low-dimensional space of postures allows for substantial variation. As for other linear shapes bent into 2D self-occluding images, we don't have a particular case in mind, (nor the data), but see no reason why our pipeline couldn't be appropriately modified.

- *If the use is limited to worms on agar, that's still a pretty wide community and essentially the tool is good for identifying nuances of turning behavior for worms on agar at scale. This suggests a question, why not just use the centroid information to perform behavioral phenotyping and studying long time behavior? Or perhaps centroid + fitting the worm to an ellipsoid to get overall angle information? What do you gain by having postural information at this scale? They attempt to address this in the last section of the results (posture-scale analysis of roaming/dwelling behavior).*

  We thank the reviewer for allowing us to clarify this fundamental point. The importance of posture is twofold: (1) posture dynamics can resolve distinct behaviors with similarly appearing centroid motions (2) posture is a closer reflection of the neuromuscular control. Previous studies have examined the genetics and neurobiology of roaming-dwelling behaviors by adopting an operational definition of low-centroid displacement as dwelling, and large centroid displacement as roaming. However, questions about the neuromuscular control strategies underlying roaming-dwelling and other long term behaviors remain open, and are difficult to address using centroid information alone. For example, it is known that when the body wall muscles are ablated, severely limiting centroid displacement, the anterior portion of the body can move normally. At the centroid level, this is not dissimilar from a dwelling state, where also the centroid displacement is limited but the head swings are normal. There are potentially many possible neuromuscular strategies for controlling the extent of centroid displacement during foraging. When and how such strategies are employed is best studied through a detailed quantification of posture dynamics. Detailed posture analysis is currently one of the only methods to gain insights into long term neuromuscular control, as most neuroimaging technologies cannot record long-time activities.

- *It is certainly impressive and a little tantalizing to have posture-level resolution in a 10 hour, 30 Hz experiment, however, I think they could have done a little more digging to say what is gained with this extremely fine grained analysis. They assess some subtleties of the difference between roaming and dwelling states which have previously been identified by simply looking at raw motion, however I don't know how salient these subtleties are, moreover the basic identification of these behavioral states is not upended by the details. Can the postural analysis make the definition of these states more robust, rather than just using centroid information to define the states and then commenting on subtle postural differences in the centroid-determined states? The most striking detail they uncover is in Fig. 6 E, but this doesn't really engage the self-occluded shapes, since these are forward and reverse travelling body waves? I don't dispute that these details are interesting, but they could be pushed further, though that may be beyond the scope of this paper.*

  We thank the reviewer for the chance to further develop our posture analysis of roaming/dwelling behavior and we have updated Fig. 6 accordingly. We have added a comparison to off-food foraging conditions Fig. 6(D, E), which provides not only a benchmark for the reversal rate analysis, but also

reveals that, contrary to common belief, dwelling worms do not exhibit an increased rate of turns. In addition, we find a complete absence of deep δ-turns in the on-food conditions, which is likely to reflect important neuromodulatory effects. We note that in addition to the tracking of coiled shapes, such a detailed analysis would not be easily accessible without WormPose, which also allowed us to resolve frequently occurring blurry frames (resulting from movement of the stage) and thus to obtain a more continuous, fast and accurate sampling of the body posture.

We agree that defining roaming and dwelling long-lived states directly from posture would be very relevant: as we've shown, low centroid speeds can results from a wide range of different posture-level movements - from increased rate of reversal and turning events (as is often postulated) to finer scale incoherent body movements that are seen as spurious reversal events at the centroid level. Since posture dynamics is more tightly coupled to the underlying neuromuscular machinery, having a fine-scale posture level description of long-lived changes in how worms explore an environment would open up new paths to the understanding of neuromodulatory pathways that drive such changes in behavior. However, such an analysis requires bridging tremendously different spatial and temporal scales: from millisecond long fine scale posture movements to minutes long coarse-grained states that correspond to different modes of exploration of the environment. We are currently working on a novel approach that enables the extraction such long lived states directly from posture-level dynamics, enabling a more robust classification of roaming and dwelling states, but we feel that such an analysis is beyond the scope of the current manuscript.

- *My last question is this, in previous work (ref 9) resolution of self-occluded shapes revealed a distinction between the delta turn and the omega turn. Since this technique allows a dramatic scale up in the number of resolvable self-intersecting states, does the larger data set shed any light on novel details of turning? Can turning behaviors be even more subtly delineated with the higher statistical resolution the technique allows?*

  In short, yes, the ability to efficiently analyze much larger sets of coiled worm posture will lead to a more nuanced understanding of turns specifically and behavior in general. WormPose has already been instrumental by providing new, fully-resolved posture for detailed analysis (Ahamed et al, *Nat Phys* **17,** 275–283 (2021)). As noted above we can already report a very strong suppression of δ-turns for worms in on-food environments relative to off-food foraging. To keep the current focus on the coiling technique however, we have reserved further analysis of the biological significance of coiled shapes for a future manuscript.

- *I would like to see terms like "generative" defined. And I would hardly call the images presented in the manuscript "Natural" images!*

  Here "generative" refers to the fact that we build a model of worm shape *probabilities* and we have added this clarification to the last paragraph of the introduction. We agree with the reviewer about the word "Natural". We have changed the heading of relevant method section to "Processing Worm Images" and we have replaced "natural" with "naturalistic" in the caption of Fig. 2.

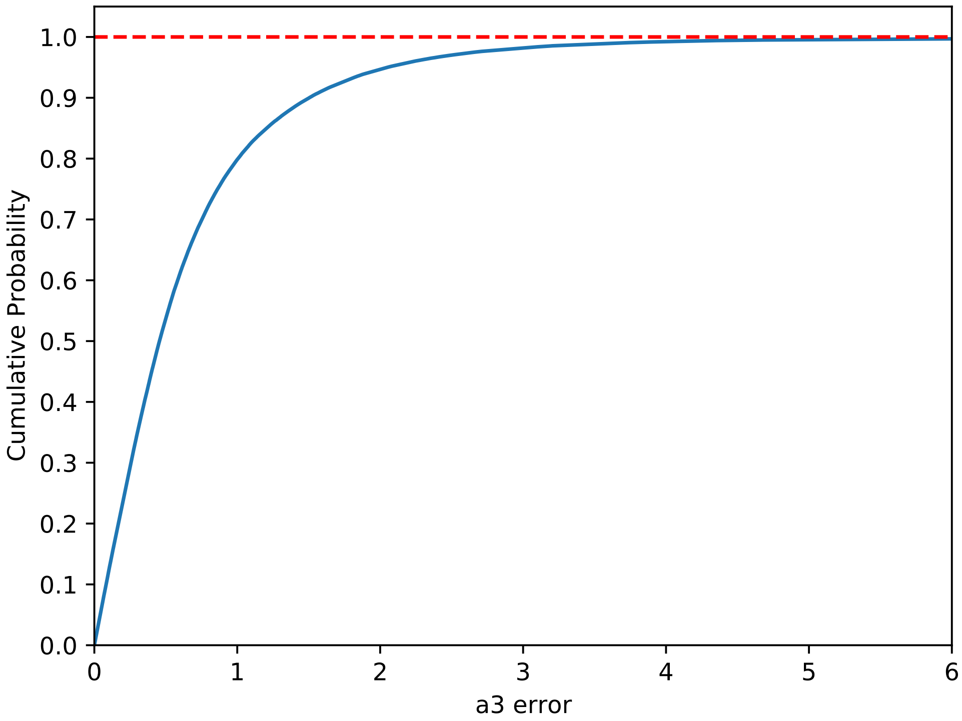

Rebuttal Figure 1: Cumulative distribution of a_3 mode errors for images in the synthetic dataset with image similarity greater than 0.7 and $|a_3| \geq 15$, thus selecting coiled shapes which pass our image similarity criterion. Only $\sim 5\%$ of these shapes have mode error $\delta a_3 > 2$, which is indicative of a less than $\sim 10\%$ error in the centerline.
